# Correlating Dose and Time of Prenatal Alcohol, Smoking, and Drug Exposure on Craniofacial Morphology: A Systematic Review

**DOI:** 10.7759/cureus.78320

**Published:** 2025-02-01

**Authors:** Anupriya Srivastava, Pradeep Raghav, Sanchit Pradhan, Amit Khera, Shehla Rafique, Pankaj Wadhwa, Ruchi Saini

**Affiliations:** 1 Orthodontics and Dentofacial Orthopaedics, Subharti Dental College, Swami Vivekanand Subharati University, Meerut, IND; 2 Public Health Dentistry, Subharti Dental College and Hospital, Swami Vivekanand Subharti University, Meerut, IND; 3 Orthodontics and Dentofacial Orthopaedics, Subharti Dental College, Swami Vivekanand Subharti University, Meerut, IND

**Keywords:** craniofacial deformity, pregnancy, prenatal alcohol exposure, prenatal drug exposure, prenatal tobacco exposure

## Abstract

The aim of this systematic review is to identify the dose and timing associated with the risk of in-utero substance exposure to craniofacial dysmorphia. This review was conducted in accordance with the Preferred Reporting Items for Systematic Reviews and Meta-Analyses (PRISMA) guidelines, considering articles written in English up to March 2024. Cochrane, Medline, and Google Scholar databases were considered. After reading all eligible studies in full text, the two reviewers discussed their results and re-read those studies for which results differed in order to find a consensus. A total of 300 studies were extracted from the databases. Eleven articles were selected in the first screening, and one more article was included after the cursory screening. This study provides data on the prevalence of growth abnormalities, as well as facial and central nervous system (CNS) anomalies in children heavily exposed to teratogens in utero, based on an unselected sample from prospectively monitored pregnancies.

## Introduction and background

Exposure to substances such as alcohol, tobacco, and drugs during pregnancy can significantly impact fetal development, particularly affecting the formation of the craniofacial complex, as outlined in the Centers for Disease Control and Prevention (CDC) guidelines [1] (2020) on alcohol use during pregnancy. This exposure often leads to craniofacial dysmorphia, characterized by abnormalities in the shape and structure of the head and face [2]. For example, prenatal alcohol exposure can result in fetal alcohol spectrum disorders (FASD), which are associated with various craniofacial defects, including microcephaly, facial hypoplasia, and ocular abnormalities [3,4]. Similarly, prenatal tobacco exposure has been associated with craniofacial defects, such as cleft lip and palate and facial asymmetry [5].

Research consistently indicates that prenatal alcohol exposure is a significant factor for craniofacial dysmorphia, with a dose-dependent increase in the severity of facial abnormalities [6]. Similarly, prenatal smoking has been correlated with an elevated risk of craniofacial defects, including cleft lip and palate. Although evidence is less robust, prenatal exposure to drugs, particularly opioids and cocaine, has been associated with craniofacial dysmorphia [7]. The mechanisms through which prenatal substance exposure affects craniofacial development are not fully understood, but they are believed to involve disruptions in genetic and environmental factors, including cellular signaling pathways and epigenetics [8]. Understanding the impact of prenatal substance exposure on craniofacial development is critical for the prevention, early intervention, and effective management of these disorders [9].

Early identification and intervention can significantly enhance outcomes for affected individuals [10]. The adverse effects of prenatal substance exposure on craniofacial development are thought to arise from disruptions in normal cellular processes, including cell proliferation, differentiation, and migration [11]. These disruptions can lead to abnormal facial morphogenesis and various craniofacial anomalies. To develop health-promoting programs aimed at reducing teratogen consumption during pregnancy, identifying which prenatal substance exposures require the most intervention is essential. Further research is needed to determine specific protocols that health promotion programs should target. Considering the observed trend of increasing craniofacial abnormalities with higher levels of embryonic substance exposure, particularly alcohol, establishing a clear threshold is difficult. Nonetheless, studies with larger sample sizes suggest that it is clinically appropriate to advise complete avoidance of teratogenic substances or, at least, minimal intake before conception. The lack of experimental data and inconsistencies in the results on the dose and timing of substance exposure highlight the need for additional research. Therefore, this systematic review aims to identify the dose and timing associated with the risk of in-utero substance exposure on craniofacial dysmorphia.

## Review

Protocol and eligibility criteria

This review was conducted per Preferred Reporting Items for Systematic Reviews and Meta-Analyses (PRISMA) [12] guidelines (CRD42024500290), considering articles written in English from 2000 to 2024. The inclusion criteria focused on studies involving patients with symptoms of prenatal exposure to alcohol, drugs, and tobacco. Exclusion criteria included animal studies; scientific papers where prenatal exposure to alcohol, tobacco, and drugs was not clearly stated; case reports; newsletters; and letters to the editor. The search strategy PICO question was defined (Table 1).

**Table 1:** The PICO question PICO=Patient/population, intervention, comparison and outcomes

PICO	
Participants	Patients with prenatal alcohol exposure, prenatal tobacco exposure and prenatal drug exposure
Intervention or exposure	Facial, dental or orthodontic findings or orofacial diagnostic methods
Comparison or control	Patients without prenatal alcohol, drug and tobacco exposure
Outcome measure(s)	Anatomic measurements of length, width or depth of craniofacial deformity and/or dental/orthodontic score(s), diagnostic method(s)
Type of studies included	Clinical studies

Study selection

Cochrane, Medline, and Google Scholar databases were considered. The search strategy was as follows: (“Pre-natal alcohol exposure” (MESH)) AND (“Pre-natal drug exposure” (MESH)) AND (“Pre-natal tobacco exposure” (MESH)) (“Face” (MESH) OR “Mouth” (MESH) OR “Oral Health” (MESH) OR “orthodontics” (MESH) OR “dental” OR “Head” (MESH) OR “palpebral fissure length” OR “diagnostic” OR “diagnose”).

All studies were screened for eligibility criteria.

Data extraction

The studies were initially reviewed by the first reviewer and classified as either “eligible” or “non-eligible.” After 15 days, the second reviewer re-evaluated the studies. This process was conducted in a blinded manner. All studies deemed eligible were read in full by both reviewers and assessed using the Quadas-2 checklist. Data on facial features in patients with prenatal exposure to alcohol, tobacco, and drugs, as well as diagnostic methods for craniofacial dysmorphia in the orofacial region, were independently extracted by the reviewers and recorded in a summary of findings table.

Following the full-text review of all eligible studies, the two reviewers discussed their results and re-read any studies where their conclusions differed to reach a consensus.

Synthesis

Synthesis of data was not possible due to inconsistent measuring methods and inhomogeneity across the studies.

Effect Measures

Effect measures were not possible due to inhomogeneity across the studies.

Bias/Quality Assessment

To minimize bias, two reviewers independently selected eligible studies for this review according to afore defined criteria and did independent full-text reading and data extraction of all included studies.

The quality of the studies was assessed using the Quadas-2 checklist. The Quadas-2 checklist is supposed to evaluate the quality of diagnostic accuracy studies.

Results

**Figure 1:**
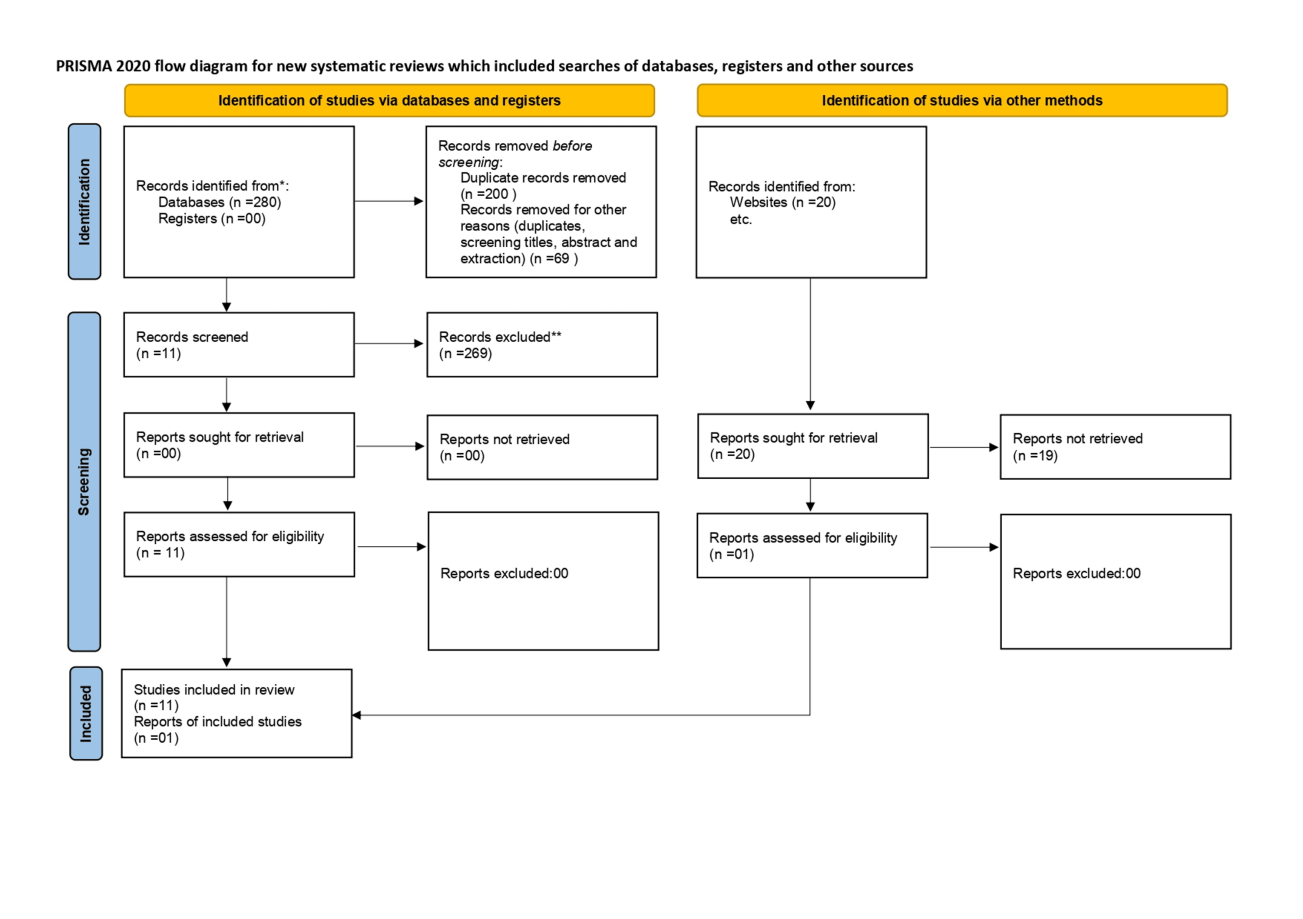
PRISMA flowchart showing the article selection procedure PRISMA = Preferred Reporting Items for Systematic Reviews and Meta-Analyses

The process of article selection is shown in Figure 1.

The list of selected studies is shown in Table 2.

**Table 2:** Brief description of the selected studies

Author	Geography	Sample year	Cases	Assessment method	Parameters taken	Conclusion
Tan et al.[13]	Australia	2019	137	Three-dimensional facial scans and principal component analysis	Pre-natal Alcohol exposure and autism spectral disorder	Developmental changes in facial exposure may be related to early alcohol exposure
Ernhart et al. [14]	USA	1987	359	Questionnaire	Pre-natal Alcohol exposure and craniofacial anomaly	Increased risk of craniofacial anomalies is associated with consumption of more than 3 ounces of alcohol at the time of conception
Muggli et al. [15]	Australia	2011-2014	415	3-dimensional craniofacial images	Pre-natal Alcohol exposure and craniofacial anomaly	Prenatal alcohol exposure, even at low levels, can influence craniofacial development.
Huget al.[16]	Missouri	1999	11	Ophthalmologic examination, magnetic resonance imaging, visual acuity test and ERG testing	Electroretinogram results and fetal alcohol syndrome	Prenatal alcohol exposure can lead to changes in retinal function as evidenced by an abnormal ERG response
Klingenberg et al.[17]	South Africa and Finland	2011	168	Geometric morphometrics	Alcohol and facial dysmorphia	Facial asymmetry with brain and neurophysiological behavioural deficits exist in patients exposed to prenatal alcohol
Kuehn et al.[18]	Chile	2012	202	Interview	Alcohol exposure and craniofacial abnormality	heavy alcohol consumption during pregnancy leads to abnormalities associated with alcohol exposure
Washington et al.[19]	Washington DC (African American mothers)	2019	180	Morphometric analysis	Alcohol, nicotine, marijuana, cocaine and heroin	Prenatal exposure to teratogens and drugs either alone or in combination with alcohol leads to amelioration of the alcohol-related facial features
Gaily et al. [20]	Helinski	1992	82	Physical anomaly score test	Pre-natal Alcohol exposure and craniofacial anomaly	As a teratogen alcohol could interfere with an originally normal developmental process causing disruption at any stage of gestation
Talens-Viscontiet al. [21]	Spain	2011	----	Immunocytochemical and real-time (RT)-polymerase chain reaction analyses	Ethanol and human embryogenic stem cell differentiation	Ethanol impairs neural progenitors survival, affects the differentiation of neural progenitors into neurons and astrocytes, affects gene expression associated with neural differentiation
Orup et al. [22], Orup et al. [23]	Boston	2003, 2014	28,13	Phenytoin, Laser light scan analysis	Phenytoin therapy and craniofacial anomaly	Widening of the philtrum and narrowing of the mouth (typical “anti-convulsant” feature) was seen in phenytoin-exposed children
Hipp et al. [24]	North Carolina	2010	6 sample well	Large-scale transcriptome analysis	Ethanol and osteogenic differentiation	Transient exposure of amniotic fluid-derived stem cells to ethanol during early differentiation enhances osteogenic differentiation of the cells.

Risk of Bias

(a) Assessment of the cohort studies: The risk of bias in the included cohort studies was assessed using the Joanna Briggs Institute (JBI) checklist for cohort studies (Table 3). The tool consists of 11 questions focusing on the selection of the study participants, exposure status, confounding, measurement of the outcome, and statistical analysis, with the option to answer "yes," indicating higher quality; "no," indicating poor quality; or "unclear." The bias risk percentage calculation is done by the amount of “Y” selected in the checklist. When “NA” was selected, this question was not considered in the calculation, according to the guidelines of the Joanna Briggs Institute. Up to 49% was considered a high risk of bias. From 50% to 70% was a moderate risk, and above 70% was a low risk of bias.

**Table 3:** Risk of bias (RoB) assessment of the cohort studies

	Autti-Ramo [20], 1992	Kuehn [18], 2012	Muggli [15], 2017
1. Were the two groups similar and recruited from the same population?	No	Yes	Yes
2. Were the exposures measured similarly to assign people to both exposed and unexposed groups?	Yes	Yes	Yes
3. Was the exposure measured in a valid and reliable way?	Yes	Yes	Yes
4. Were confounding factors identified?	Yes	Yes	Yes
5. Were strategies to deal with confounding factors stated?	Yes	Yes	Yes
6. Were the groups/participants free of the outcome at the start of the study (or at the moment of exposure)?	Yes	Yes	Yes
7. Were the outcomes measured in a valid and reliable way?	Yes	Yes	Yes
8. Was the follow up time reported and sufficient to be long enough for outcomes to occur?	Yes	Yes	Yes
9. Was follow up complete, and if not, were the reasons to loss to follow up described and explored?	No	No	Yes
10. Were strategies to address incomplete follow up utilized?	No	No	Yes
11. Was appropriate statistical analysis used?	Yes	Yes	Yes
% Yes	72.7	81.8	100
RoB	Low	Low	Low

The study quality and presence of biases among cohort studies were determined using the JBI Critical Appraisal Checklist for cohort studies. All the studies that were assessed using the checklist for possible bias showed a low risk of bias.

(b) Assessment of in-vitro studies: The quality of the selected studies was individually assessed. In accordance with the Quality Assessment Tool For In Vitro Studies (QUIN Tool), the following 12 different criteria were considered (Table 4): clearly stated aims/objectives, detailed explanation of sample size calculation, detailed explanation of sampling technique, details of the comparison group, detailed explanation of methodology, operator details, randomization, method of measurement of outcome, outcome assessor details, blinding, statistical analysis, and presentation of results. Each criterion can be adequately specified (score = 2), not adequately specified (score = 1), not specified (score = 0), or not applicable (NA). Then, the 12 scores are added to obtain the final score for each study. In the end, the result obtained is used to grade every single study as high, medium, or low risk (>70% = low risk of bias, 50%-70% = medium risk of bias, and <50% = high risk of bias) by using the following formula: Final score = (Total score × 100)/(2 × number of criteria applicable).

**Table 4:** Risk of bias assessment of In-vitro studies Score: adequately specified: 2 points; inadequately specified: 1 point; not specified: 0 points; not applicable: NA. BIAS: low risk > 70%; medium risk between 70% and 50%; high risk < 50%

Criteria	Hipp [23], 2010	Talens-Visconti [21], 2011
Clearly stated aims/objectives	2	2
Detailed explanation of sample size calculation	0	0
Detailed explanation of the sampling technique	NA	NA
Details of the comparison group	2	2
A detailed explanation of the methodology	2	2
Operator details	0	0
Randomization	NA	NA
Method of measurement of outcome	2	2
Outcome assessor details	0	0
Blinding	0	0
Statistical analysis	2	2
Presentation of results	2	2
Total score	12	12
Total score (in %)	60	60
Risk of bias	Moderate	Moderate

The risk of bias of included studies is summarized in the above-mentioned table. Both the studies included presented with a moderate risk of bias. Aims and objectives were mentioned in all the studies; however, sample size estimation was not mentioned in any studies. All the studies adequately presented details of the comparison group and an explanation of the methodology. Outcome assessor details were not reported in any of the studies. Results were presented appropriately, and statistical analysis was reported adequately in both studies.

(c) Assessment of the cross-sectional studies: The risk of bias in the included cross-sectional studies was assessed using the JBI checklist for cross-sectional studies (Table 5). The tool consists of eight questions focusing on the inclusion of the participants, exposure status, confounding, measurement of the outcome, and statistical analysis, with the option to answer "yes," indicating higher quality; "no," indicating poor quality; or "unclear." The bias risk percentage calculation is done by the amount of “Y” selected in the checklist. When “NA” was selected, this question was not considered in the calculation, according to the guidelines of the JBI. Up to 49% was considered a high risk of bias. From 50% to 70% was a moderate risk, and above 70% was a low risk of bias.

**Table 5:** Risk of bias assessment of the cross-sectional studies

	Ernhart [14], 1987	Hug [16], 2010	Klingenberg [17], 2010	Orup [22], 2003	Orup [ 23], 2014	Tan [13], 2020	Washington [19], 2019
1. Were the criteria for inclusion in the sample clearly defined?	Yes	Yes	Yes	Yes	Yes	Yes	Yes
2. Were the study subjects and the setting described in detail?	Yes	No	Yes	Yes	Yes	Yes	Yes
3. Was the exposure measured in a valid and reliable way?	Yes	Unclear	Unclear	Unclear	Yes	Yes	Yes
4. Were objective, standard criteria used for measurement of the condition?	Yes	Yes	Yes	Yes	Yes	Yes	Yes
5. Were confounding factors identified?	Yes	No	Yes	No	No	Yes	No
6. Were strategies to deal with confounding factors stated?	Yes	No	Yes	No	No	Yes	No
7. Were the outcomes measured in a valid and reliable way?	Yes	Yes	Yes	Yes	Yes	Yes	Yes
8. Was appropriate statistical analysis used?	Yes	Yes	Yes	Yes	Yes	Yes	Yes
% Yes	100	50	100	62.5	75	100	75
Risk	Low	Moderate	Low	Moderate	Low	Low	Low

The study quality and presence of biases among cross-sectional studies were determined using the JBI Critical Appraisal Checklist for cross-sectional studies. Seven studies that were assessed using the checklist for possible bias showed a low risk of bias, whereas two studies showed a moderate risk of bias.

Discussion

This systematic review aims to gather and summarize all available data on the effects of prenatal maternal exposure to teratogens -- including drugs, alcohol, and smoking -- on craniofacial dysmorphism in neonates. The review focuses exclusively on human studies, as findings from animal models, although informative regarding potential dose-response relationships, do not fully translate to human outcomes [13].

A total of 12 articles were selected from a literature search conducted across various databases. Among these, nine studies examined the effects of alcohol on craniofacial morphology and neural/stem cell differentiation, two studies focused on phenytoin's effects, and one study investigated the combined impact of alcohol, cocaine, and marijuana.

Most of the selected studies were retrospective, relying on questionnaires and interviews. Some studies were conducted in vitro, while others utilized methodologies such as morphometric analysis, cephalometric analysis, immunocytochemistry, and transcription studies.

Ernhart et al. [14]. conducted a cohort study involving 359 neonates, concluding that consuming more than three ounces of absolute alcohol at the time of conception can lead to craniofacial anatomical abnormalities. Muggli et al. [15] explored the dose, frequency, and timing of prenatal alcohol exposure and its effects on the craniofacial phenotype in 12-month-old children, suggesting that even low doses of alcohol can influence cranial deformities - particularly in the midface, nose, lips, and eyes.

However, some studies, such as those by Lumley et al. [24], did not observe any anomalies at low levels of alcohol exposure. Hug et al. [16] conducted a study involving ophthalmologic examinations, magnetic resonance imaging, visual acuity tests, and ERG on patients with fetal alcohol syndrome, finding that, alongside neurological issues, ophthalmic conditions such as optic nerve hypoplasia, ptosis, and strabismus were present. Similarly, a study by Klingenberg et al. [17] demonstrated a connection between facial asymmetry and other brain-related functions, as well as neurophysiological and behavioral deficits due to prenatal alcohol exposure.

Kuehn et al. [18] studied pregnant women regarding the intensity and frequency of alcohol intake, concluding that both binge drinking and total intake significantly contribute to craniofacial deformity development. Alcohol, whether alone or in combination with other substances, acts as a potent teratogen. A morphometric analysis conducted by Washington et al. [19] on 180 African American neonates showed that exposure to nicotine, marijuana, cocaine, and heroin, with or without, could exacerbate alcohol-related facial features. The critical period for teratogen exposure is during the early first trimester, or embryonic period.

Studies by Autti-Ramo et al. [20], Tan et al. [13], and Raqueĺns-Visconti et al. [21] indicate that such exposure can lead to impaired brain development, hindered head growth, and a relatively long midface. Two studies by Orup et al. [22,23] examined the effects of in-utero phenytoin exposure, both monotherapy and polytherapy. One study using cephalometric, hand-wrist, and panoramic radiographs identified craniofacial deviations, such as decreased height and length of the maxilla and mandible, as well as a reduction in cranial width. The second study employed 3D laser scanning, revealing significant changes in facial soft tissue, including widening of the philtrum and narrowing of the mouth.

In-vitro studies using human embryonic stem cells by Taléns-Visconti et al. [21] and Hipp et al. [25] demonstrated that ethanol affects epigenetic mechanisms, including DNA methylation and histone modifications. It also impairs neural progenitor survival and disrupts the differentiation of neural progenitors into neurons and astrocytes, affecting various cytoskeletal components and the expression of genes associated with neural differentiation.

Emerging technologies, such as virtual reality (VR), augmented reality (AR), and the metaverse, offer promising avenues for addressing craniofacial dysmorphia linked to prenatal substance exposure. VR and AR can assist in early diagnosis, intervention, and rehabilitation, while the metaverse could enhance medical education and remote consultations. Blockchain technology can ensure secure patient data management. Digital therapeutics may offer solutions for vaping cessation among pregnant women. These technologies, integrated with health care, could significantly improve prevention and treatment outcomes.

## Conclusions

Our study provides critical data on the prevalence of growth abnormalities, as well as facial and central nervous system (CNS) anomalies in children heavily exposed to teratogens in utero, based on an unselected sample from prospectively monitored pregnancies.

We observed aspects of a craniofacial phenotype with almost any level of prenatal alcohol exposure, but synergistic effects of long-term alcohol exposure with smoking or drug exposure showed more serious effects. These findings support the conclusion that, for women who are, or may become pregnant, avoiding alcohol is the safest option, and it is indicated that women should be counseled about the significant risk factors associated with both binge drinking and total alcohol intake during pregnancy.
